# Seasonal Filarial Infections and Their Black Fly Vectors in Chiang Mai Province, Northern Thailand

**DOI:** 10.3390/pathogens9060512

**Published:** 2020-06-25

**Authors:** Kittipat Aupalee, Atiporn Saeung, Wichai Srisuka, Masako Fukuda, Adrian Streit, Hiroyuki Takaoka

**Affiliations:** 1 Graduate Doctoral Degree Program in Parasitology, Faculty of Medicine, Chiang Mai University, Chiang Mai 50200, Thailand; kittipat.aupalee@gmail.com; 2 Department of Parasitology, Faculty of Medicine, Chiang Mai University, Chiang Mai 50200, Thailand; 3 Entomology Section, Queen Sirikit Botanic Garden, P.O. Box 7, Chiang Mai 50180, Thailand; wsrisuka@gmail.com; 4 Institute for Research Promotion, Oita University, Idaigaoka 1-1, Hasama, Yufu City, Oita 879-5593, Japan; mfukuda@oita-u.ac.jp; 5 Department of Integrative Evolutionary Biology, Max Planck Institute for Developmental Biology, Tübingen, 72076 Baden-Württemberg, Germany; adrian.streit@tuebingen.mpg.de; 6 Tropical Infectious Diseases Research and Education Centre (TIDREC), University of Malaya, Kuala Lumpur 50603, Malaysia; takaoka@oita-u.ac.jp

**Keywords:** *Simulium*, black fly, *Onchocerca* spp., zoonotic onchocerciasis, *cox1*, 12S rRNA, 18S rRNA

## Abstract

The transmission of zoonotic filarial parasites by black flies has so far been reported in the Chiang Mai and Tak provinces, Thailand, and the bites of these infected black flies can cause a rare disease—human zoonotic onchocerciasis. However, species identification of the filarial parasites and their black fly vectors in the Chiang Mai province were previously only based on a morphotaxonomic analysis. In this study, a combined approach of morphotaxonomic and molecular analyses (mitochondrial *cox1*, 12S rRNA, and nuclear 18S rRNA (*SSU* HVR-I) genes) was used to clarify the natural filarial infections in female black flies collected by using human and swine baits from two study areas (Ban Lek and Ban Pang Dang) in the Chiang Mai province from March 2018 to January 2019. A total of 805 and 4597 adult females, belonging to seven and nine black fly taxa, were collected from Ban Lek and Ban Pang Dang, respectively. At Ban Lek, four of the 309 adult females of *Simulium nigrogilvum* were positive for *Onchocerca* species type I in the hot and rainy seasons. At Ban Pang Dang, five unknown filarial larvae (belonging to the same new species) were detected in *Simulium* sp. in the *S. varicorne* species-group and in three species in the *S*. *asakoae* species-group in all seasons, and three non-filarial larvae of three different taxa were also found in three females of the *S. asakoae* species-group. This study is the first to molecularly identify new filarial species and their vector black fly species in Thailand.

## 1. Introduction

Black flies (Diptera: Simuliidae) are a group of small hematophagous insects which vector several pathogens that are responsible for animal and human diseases, particularly human onchocerciasis (river blindness) caused by the parasitic worm *Onchocerca volvulus* [1]. Several black fly species have also been reported to vector filarial worms of the genus *Onchocerca* that cause zoonotic onchocerciasis in humans. This infection is generally caused by a single immature adult worm of animal origin [2]. So far, 37 human cases of zoonotic onchocerciasis have been reported globally, including in Europe, North America, North Africa, and East Asia [3]. Recently, the 38th case, a male infected in his eye with *O*. *jakutensis*, which is a natural parasite of red deer, was reported in Poland [4].

Until now, a total of 35 valid *Onchocerca* species have been reported worldwide, including a newly described species, *O. borneensis*, from Malaysia [5,6]. Furthermore, Uni et al. [6] raised *O*. *dewittei japonica* to the species level (the 36th) as *O*. *japonica*, based on morphological and molecular analyses. Among these *Onchocerca* spp., five, namely *O*. *gutturosa*, *O*. *cervicalis*, *O*. *jakutensis*, *O. lupi*, and *O. japonica*, were identified as the causative agents of the above-mentioned zoonotic onchocerciasis [3,6].

In Thailand, two black fly species, *Simulium chumpornense* and *Simulium asakoae* s. l., were recently reported as potential vectors of the avian blood protozoa *Leucocytozoon* and *Trypanosoma* [7,8,9]. Even though neither human onchocerciasis nor zoonotic onchocerciasis has been recorded in Thailand, three human-biting black fly species, namely *Simulium nodosum*, *S*. *asakoae* s. l., and *Simulium nigrogilvum*, were found to be natural vectors of three types of filarial worms in the Chiang Mai province, in the northern region of Thailand [10,11,12]. However, natural filarial infections in black flies in the areas of the Chiang Mai province have not been monitored and examined for over a decade. Previous studies were solely based on the morphological characters of the recovered larvae, and molecular species identification is currently missing [10,11,12].

In order to explore the existence of natural filarial infections in black fly species previously regarded as vectors of unknown filarial worms, we recently conducted a preliminary study in the Fang district, Chiang Mai province, northern Thailand. We determined natural filarial infections by dissecting black flies and searching for any stages of filarial larvae. The result revealed, for the first time, that two of 52 adult females of *S. nigrogilvum* dissected were naturally infected with third-stage larvae (L_3_, infective larvae) of unknown filarial worms, which were morphologically and molecularly identified as *Onchocerca* sp. type I—an unnamed bovine filarial worm isolated from *Simulium bidentatum* in Japan [13,14,15]. A similar detection of *O.* sp. type I in *S. nigrogilvum* was recently reported in the Tak province, western Thailand, by Saeung et al. [16].

In the current study, to effectively plan public health strategies for dealing with the transmission of zoonotic filariae to humans, we attempted to gather additional information about the filarial parasites and their black fly vectors present in Thailand, as well as their geographical distribution. Therefore, we examined adult female flies to determine their natural filarial infections. Furthermore, we morphologically and molecularly analysed the infected black flies and filarial larvae recovered to elucidate their taxonomic position.

## 2. Results

### 2.1. Black Fly Species Collected and Seasonal Abundance

#### 2.1.1. Ban Lek (BL)

The total numbers of female black flies collected in each season are shown in Table 1. A total of 805 females of seven black fly taxa were captured during the study period. *Simulium chumpornense* (39.4%), *S*. *nigrogilvum* (38.3%), and *Simulium* spp. in the *S*. *asakoae* species-group (18.4%) were relatively abundant, whereas four other *S*. spp. were minor (3.9%) (Table 1)*. Simulium chumpornense* was only captured in the hot season (March), but then in a very high number, while *S. nigrogilvum*, species in the *S*. *asakoae* species-group, and species in the *S. striatum* species-group were collected throughout the year, with a peak in the hot season.

#### 2.1.2. Ban Pang Dang (BPD)

The total numbers of female black flies collected in each season are shown in Table 2. Nine black fly taxa were captured throughout the year. Among the 4597 flies collected, the most abundant species was *S*. spp. in the *S*. *asakoae* species-group (50.7%), followed by *S. chumpornense* (48.9%) and seven other taxa (0.4%) (Table 2). *Simulium chumpornense* was captured in a very high number, but only in the hot season (March), while species in the *S*. *asakoae* species-group were collected throughout the year, with a peak in the dry-cool season.

### 2.2. Natural Filarial Infections and Morphological Identification of Recovered Larvae

#### 2.2.1. Ban Lek

**Figure 1:**
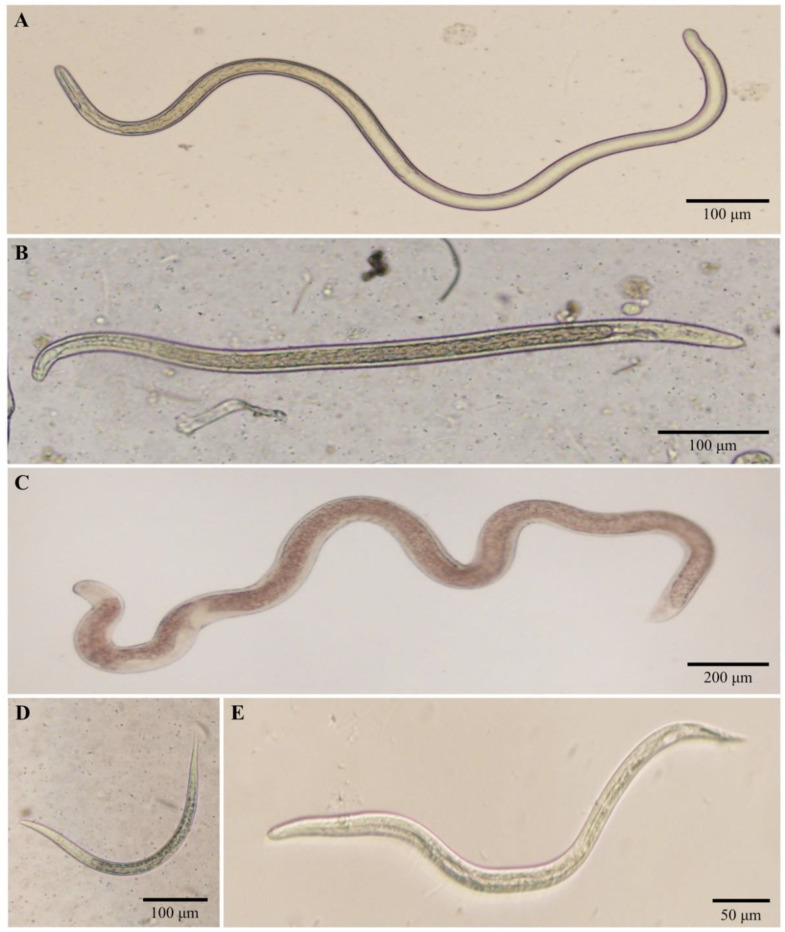
Filarial and non-filarial larvae recovered from thoraxes of adult female black flies. (**A**) L_3_ larva of *Onchocerca* sp. type I recovered from *S. nigrogilvum*. (**B**) L_3_ larva of an unknown filarial species recovered from three species in the *S*. *asakoae* species-group and *Simulium* sp. in the *S. varicorne* species-group. (**C**–**E**) L_3_ larvae of mermithid, rhabditida, and ascaridoid nematodes recovered from species in the *S. asakoae* species-group, respectively.

Natural filarial infections were only found in four females of *S. nigrogilvum*. Three out of the 187 *S. nigrogilvum* collected in the hot season, one out of the 82 collected in the rainy season, and none out of the 40 collected in the dry-cool season were naturally infected with one or two infective larvae (L_3_), each in the thorax (Table 1, Figure 1A), resulting in an overall infection rate of *S. nigrogilvum* of 1.3%. The sizes of these larvae were 1027.1–1339.5 µm long by 25.6–29.6 µm wide (Table 3). All six recovered larvae were morphologically similar to one another and were identified as belonging to the genus *Onchocerca* [17]. These larvae were most similar in size (width and length) and morphology to *O.* sp. type I [13,16].

#### 2.2.2. Ban Pang Dang

In total, nine female black flies (*S*. sp. (*n* = 1) and *S. asakoae* species-group (*n* = 8)) were infected with unknown nematode larvae. Ten larvae were recovered from these infected flies and tentatively identified as filarial nematodes (*n* = 5), non-filarial nematodes (*n* = 3), and unidentified nematodes (*n* = 2), based on their morphological characters. Natural infections with filarial larvae were found over the year in two black fly species, including one in the *S.* sp. (collected in the hot season, L_1_) and three in the *S. asakoae* species-group. Of the *S. asakoae* species-group, one of 635 collected in the rainy season and two of 1175 collected in the dry-cool season were infected (Table 2). One or two infective larvae (L_3_) were recovered from the thoraxes of each infected female (Figure 1B). Furthermore, three females of the *S. asakoae* species-group collected in the rainy and dry-cool seasons were also naturally infected with three non-filarial larvae. One of these larvae was morphologically identified as a mermithid nematode (Figure 1C) and the other two larvae were morphologically identified as two different, unknown non-filarial nematodes (Figure 1D,E). For two larvae recovered from flies of the *S. asakoae* species-group (one collected in the hot season (June: Mf) and one collected in the rainy season (November, L_1_)), the species status could not be evaluated due to damage, which made morphological observations difficult and prevented molecular analysis (Table 3).

### 2.3. Molecular Identification of Recovered Larvae

#### 2.3.1. Ban Lek

**Figure 2:**
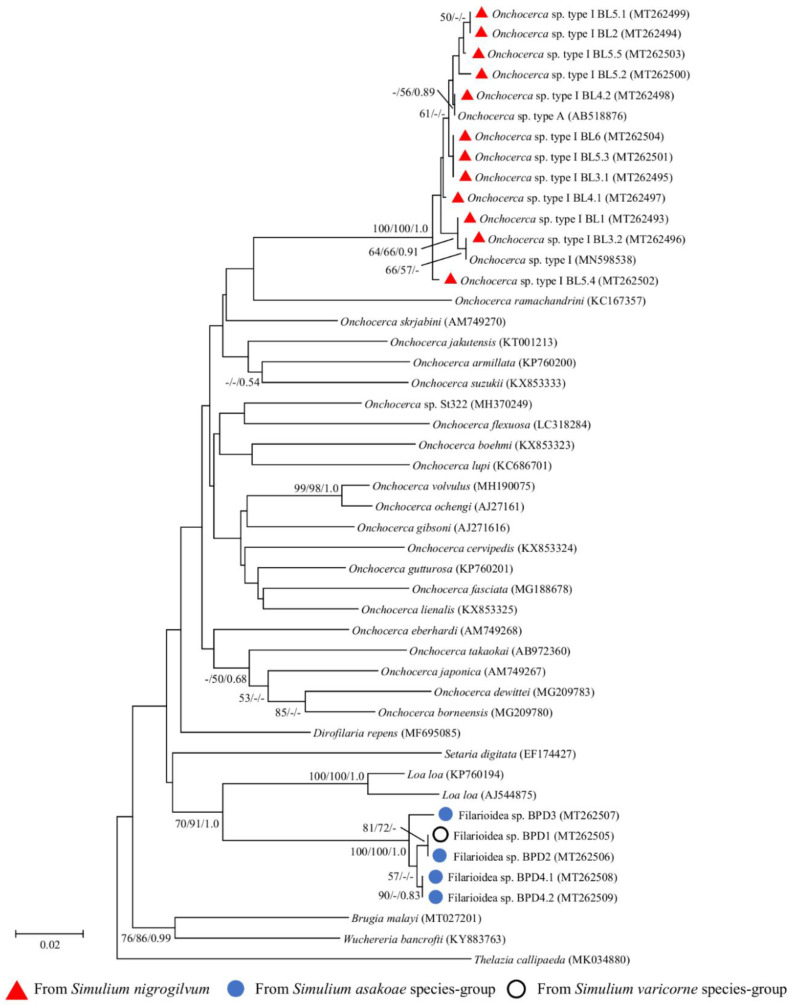
Neighbor-joining tree of *Onchocerca* spp. and unknown filarial larvae based on the *cox1* gene sequences. Bootstrap values and posterior probabilities (neighbor-joining (NJ)/maximum-likelihood (ML)/Bayesian inference (BI)) are shown above or near the branches. A dash indicates that support values are less than 50% (for NJ and ML) or 0.50 (for BI). The scale bar represents 0.02 substitutions per nucleotide position. Red triangles, blue circles, and white circle with a black border before each specimen designate the specimens obtained from this study and also indicate the vector blackfly species.

**Figure 3:**
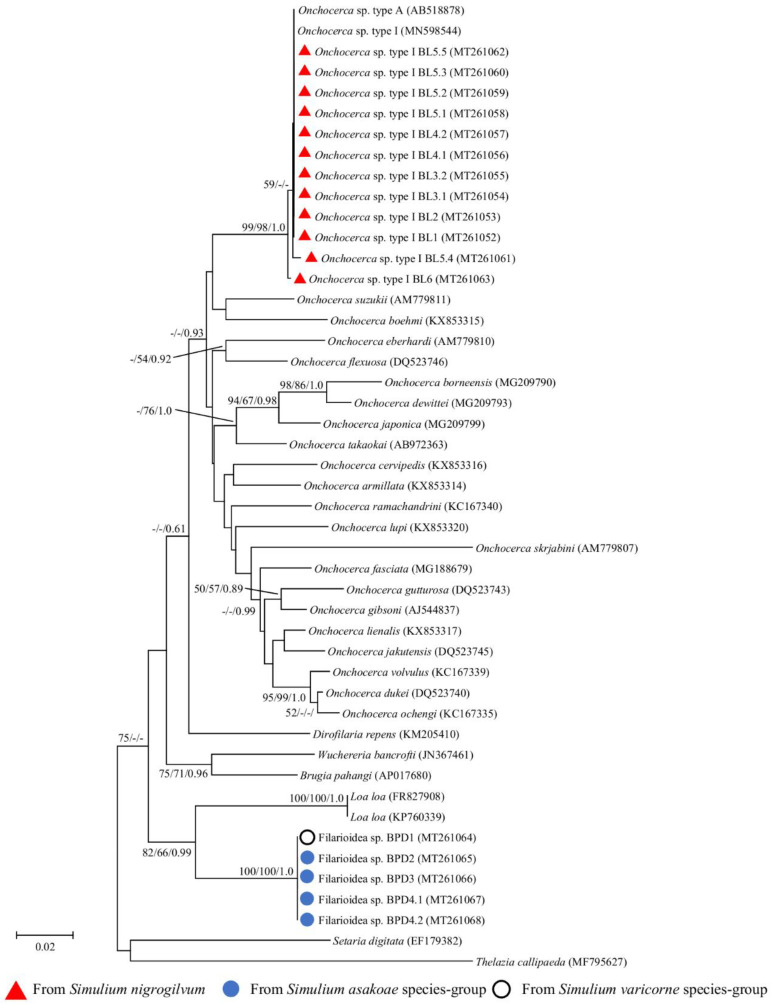
Neighbor-joining tree of *Onchocerca* spp. and unknown filarial larvae based on the 12S rRNA gene sequences. Bootstrap values and posterior probabilities (NJ/ML/BI) are shown above or near the branches. A dash indicates that support values are less than 50% (for NJ and ML) or 0.50 (for BI). The scale bar represents 0.02 substitutions per nucleotide position. Red triangles, blue circles, and white circle with a black border before each specimen designate the specimens obtained from this study and also indicate the vector blackfly species.

Molecular species identification based on *cox1* and 12S rRNA gene sequences was performed for all thirteen infective larvae (including seven larvae from the preliminary study), and twelve of them were successfully amplified. The sequence fragments generated were 649 and 470–471 bp long for the *cox1* and 12S rRNA genes, respectively. Phylogenetic analyses revealed that *O*. sp. type I of our study formed a monophyletic clade with *O*. sp. type A recovered from *S. bidentatum* in Japan and *O*. sp. type I recovered from *S. nigrogilvum* in western Thailand (Figure 2 and Figure 3). The very small genetic distance (K2P, *cox1*: 0.00–0.89%; 12S rRNA: 0.00–0.27%) between the *O*. sp. type I isolated in this study and *O*. sp. type I (= type A) retrieved from GenBank strongly suggested that they were the same *O*. sp. Compared with other *O*. spp., our *O*. sp. type I sequences showed 9.43–12.96% and 5.34–10.80% sequence difference for *cox1* and 12S rRNA genes, respectively.

#### 2.3.2. Ban Pang Dang

The *cox1* and 12S rRNA genes of all five unknown filarial larvae were successfully amplified. The sequence fragments generated were 649 and 464 bp long for the *cox1* and 12S rRNA genes, respectively. Phylogenetic analyses based on both mitochondrial genes showed that all five larvae were clustered together and formed a monophyletic clade with high bootstrap support (Figure 2 and Figure 3). They were placed as a sister group to *Loa loa*. Additionally, the sequence analysis of both genes using BLAST revealed that all larvae were closest to filarioid nematodes, i.e., *Dirofilaria repens, Wuchereria bancrafti, Brugia malyai*, and *Loa loa*, with >89% sequence identity (Table S1). However, the genetic distance between our unknown filarioid nematodes and all filarioid nematodes for which the corresponding information was present in the databases was still rather high (K2P, *cox1*: 12.03–21.53%; 12S rRNA: 8.70–14.40%), suggesting that our unknown larvae represent a new species.

**Figure 4:**
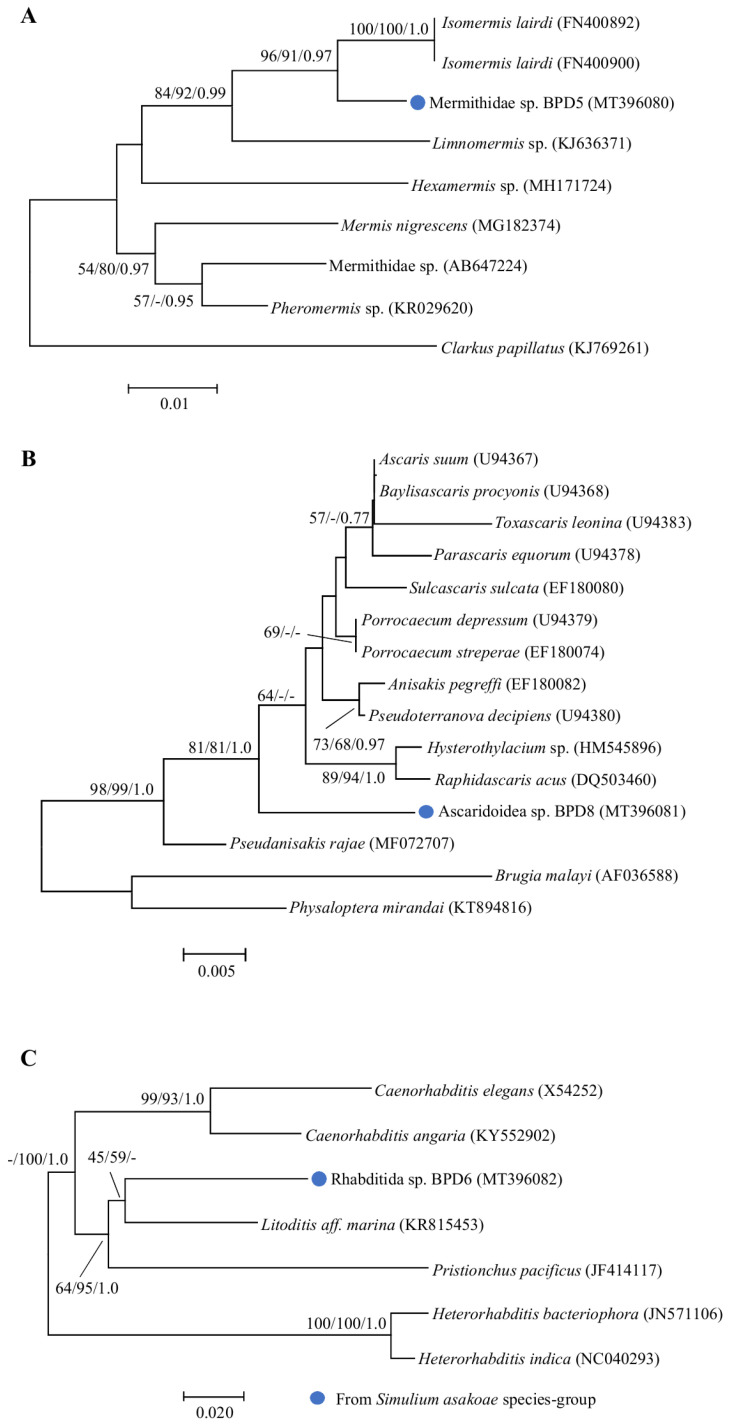
Neighbor-joining trees of non-filarial nematodes. Bootstrap values and posterior probabilities (NJ/ML/BI) are shown above or near the branches. A dash indicates that support values are less than 50% (for NJ and ML) or 0.50 (for BI). Blue circles before each specimen designate the specimens obtained from this study and also indicate the vector blackfly species. (**A**) An 18S rRNA tree of mermithid nematodes. (**B**) An 18S rRNA tree of ascaridoid nematodes. (**C**) An 12S rRNA tree of rhabditida nematodes.

For the three non-filarial larvae recovered from three flies in the *S. asakoae* species-group, an analysis of the 18S rRNA sequence using BLAST and phylogenetic analyses revealed that they were indeed three different species of non-filarial nematodes. The first of the unknown nematodes resembling mermithid nematodes was highly similar (97% sequence identity) to *Isomermis lairdi*, which is a known mermithid parasite of simuliids (Table S1). However, although the genetic differentiation between this mermithid nematode and *I. lairdi* was relatively low (1.84%), in the phylogentic analyses, this mermithid nematode was separated with high bootstrap support from the published *I. lairdi* specimens, suggesting that it might be a different species (Figure 4A). The second of the unknown nematodes was highly similar to several ascaridoid nematode species, for example, *Ascaris suum, Porrocaecum streperae*, and *Pseudoterranova decipiens*, with >97% sequence identity in the 18S rRNA gene sequence (Table S1). The genetic divergence was moderate (1.96–3.21%) and in the phylogenetic analyses, it was well-separated from all other ascaridoid nematode species retrieved from databases, suggesting that it also might be an unknown species (Figure 4B). The third of the unknown nematodes was genetically close to *Litoditis* aff. *marina*, with 90% sequence identity in the 12S rRNA gene sequence (Table S1). With a genetic distance of 10.41–17.58% between this rhabditida nemotode and its most closely related known species, it is likely that this worm also represents a novel species (Figure 4C).

### 2.4. Molecular Identification of Infected Black Flies

**Figure 5:**
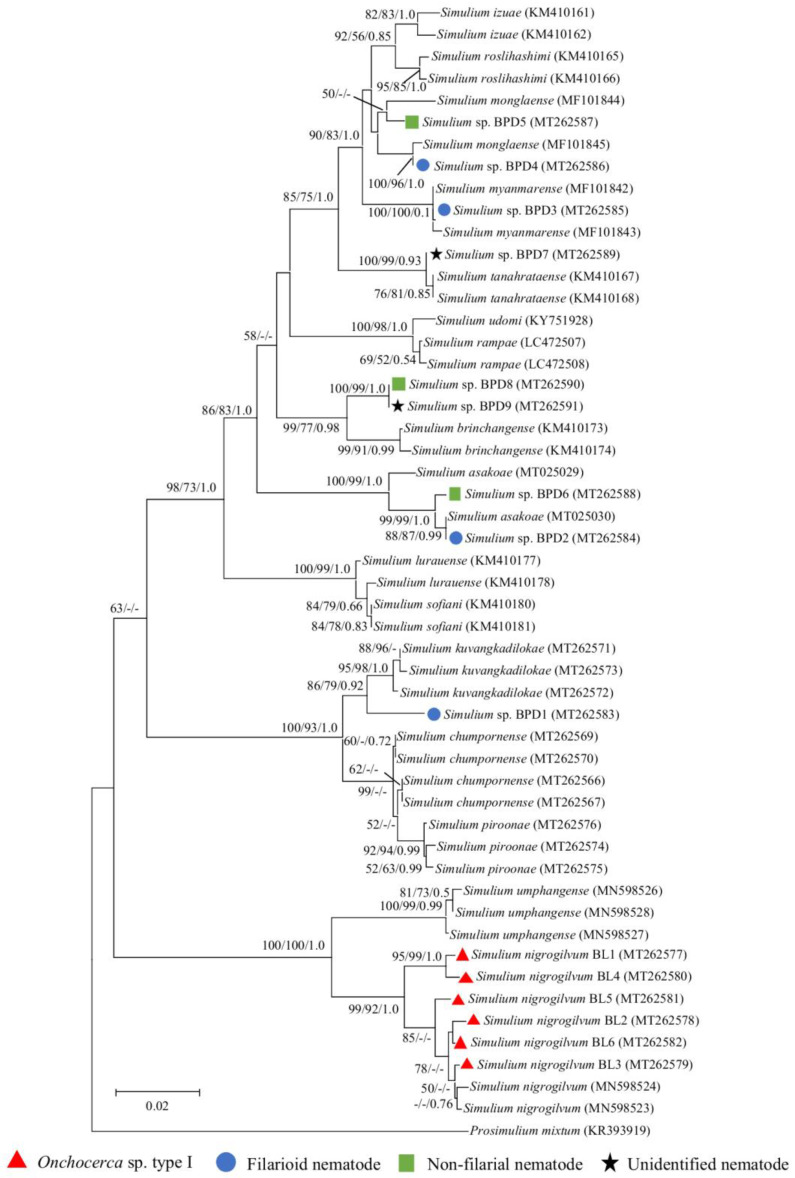
Neighbor-joining tree of infected black flies based on *cox1* gene sequences. Bootstrap values and posterior probabilities (NJ/ML/BI) are shown above or near the branches. A dash indicates that support values are less than 50% (for NJ and ML) or 0.50 (for BI). The scale bar represents 0.02 substitutions per nucleotide position. Red triangles, blue circles, green squares, and black stars before each specimen designate the specimens obtained from this study and also indicate the species/group of nematode larvae recovered.

To confirm the exact species of infected black flies, molecular species identification based on *cox1* gene sequences was performed. All 13 infected black flies (*S. nigrogilvum* (*n* = 4), species in the *S*. *asakoae* species-group (*n* = 8), and *S.* sp. in the *S. varicorne* species-group (*n* = 1)) were successfully amplified with a length of 658 bp. All six infected flies that had been morphologically identified as *S. nigrogilvum* formed a monophyletic group together with the *S. nigrogilvum* reference sequences retrieved from the database. This supported that they all were truly *S. nigrogilvum*. The *S. nigrogilvum* group was clearly separated from the closely related species *Simulium umphangense* (genetic distances 5.17–6.74%) (Figure 5). The fly morphologically classified as unknown *S.* sp. in the *S*. *varicorne* species-group (BPD1) turned out to be genetically related to three members of the *S. chumpornense* subgroup, but was clearly separated from them (Figure 5). *Simulium* sp. (BPD1) was distinguished from S. *chumpornense* by 3.11–3.30%, *Simulium kuvangkadilokae* by 1.99–2.36%, and *Simulium piroonae* by 3.49–3.68%. Based on these relatively high genetic distances between *S.* sp. and its most closely related species, this black fly is most likely a new species.

For the eight flies morphologically assigned to the *S*. *asakoae* species-group, the *cox1* sequences were compared with 11 other known species within the *S. asakoae* species-group. Based on phylogenetic analyses and comparisons of genetic distances, six flies were tentatively identified as belonging to four known species of the *S. asakoae* species-group, namely *S. asakoae* (BPD2 and BPD6), *Simulium monglaense* (BPD4 and BPD5), *Simulium myanmarense* (BPD3), and *Simulium tanahrataense* (BPD7) (Table S2, Figure 5). Two specimens (BPD8 and BPD9) most likely represent a new species in the *S. asakoae* species-group, as they formed a strongly supported monophyletic clade with a moderate to high genetic distance when compared to other known species (2.35–8.33%) (Table S2, Figure 5).

## 3. Discussion

Black flies are well-known to play the most important role in the transmission of zoonotic onchocerciasis to humans [2]. However, information on the transmission of this disease by black flies in Thailand is very limited. This study is the first to clarify the species status of filarial parasites and their black fly vectors in the Chiang Mai province, northern Thailand, using a combined approach of morphotaxonomic and molecular analyses. Information obtained from this study will be useful in planning the preventive measurements of human zoonotic onchocerciasis that may occur in the country in the future. In addition, we collected black fly samples at two study sites which have different elevations. Our findings are in agreement with a previous study conducted by Srisuka et al. [18], who reported that the elevation influences the presence or absence of particular black fly species in Thailand. On the other hand, we also found examples for non-altitude-dependent species. For example, the *S*. *asakoae* species-group was widely distributed from low elevations to 1600 m in high mountains. In the first study area (Ban Lek, high elevation), seven black fly taxa were collected. Among these, the *S*. *asakoae* species-group, *S*. *chamlongi*, the *S. doipuiense* complex, and *S*. *nigrogilvum* have been reported as human-biting species in previous studies [11,19,20,21]. The *Simulium doipuiense* complex was only collected from high elevations above sea level, similar to previous studies by Ittiponpanya [19] and Pramual et al. [21], who also found this species at high elevations (range between 1200 and 1600 m). *Simulium chumpornense* and *S*. *nigrogilvum* were the predominant taxa, consistent with Srisuka et al. [18], who noted that a large number of adult female flies of these species were collected, although no larvae and pupae were found in this area.

To date, in Thailand, only one previously undescribed *Onchocerca* sp. (*O*. sp. type I) has been confirmed to infect *S*. *nigrogilvum* based on morphological characterization and DNA sequence analyses [16]. Similarly, our study revealed that *S*. *nigrogilvum*, collected at Ban Lek is a natural vector of *O*. sp. type I, which has previously been isolated from *S*. *bidentatum* in Japan and *S*. *nigrogilvum* in western Thailand [15,16]. *Simulium nigrogilvum* has been regarded as a natural vector for possible zoonotic onchocerciasis by *O*. sp. type I because it harbors the infective stage (L_3_) of the disease agent and prefers to feed on humans (anthropophily) [9]. Furthermore, *O*. sp. type I has a broad geographical distribution, as it has been found from Thailand to Kyushu and Honshu in Japan [15,16]. In Thailand, cattle or water buffaloes (or both) are suspected to be reservoir hosts of this *O*. sp., owing to the fact that other ungulates were absent in and around the study area [13]. Natural filarial infections were found throughout the year, except for the dry-cool season, in which only a very low number of black flies were collected. The infection rate of *O.* sp. type I in *S. nigrogilvum* was slightly higher (1.3%) than in a previous study (0.8%) conducted in western Thailand [16].

In the second area (Ban Pang Dang, low elevation), among the nine black fly taxa captured, the *S*. *asakoae* species-group was the predominant one. This finding was consistent with previous reports by Takaoka et al. [11] and Ishii et al. [12], who studied the daily biting activity patterns and natural filarial infections of adult black flies in Doi Saket district, Chiang Mai province. In this area, natural infections were observed in all seasons, but only the *S*. *asakoae* species-group and *S*. sp. were found to be natural vectors of filarial and/or non-filarial nematodes. The pig farm in this area may influence (1) the species composition of black flies, if some black fly species show a preference for pigs as a blood-meal host, and (2) the species of *Onchocerca* larvae carried by black flies. However, there is no information about *Onchocerca* infections in pigs in Thailand or other countries. Hence, further studies are needed to elucidate whether the filarial larvae and/or the non-filarial larvae found in black flies in this study affect livestock or local wildlife.

In this study, based on DNA sequence analyses of two mitochondrial genes, one species of unknown filarial nematode was found to infect three species in the *S*. *asakoae* species-group and *S*. sp. in the *S*. *varicorne* species-group. We followed the criteria for distinguishing different species of filarioid nematodes (*cox1* interspecific distances > 4.8%) suggested by Ferri et al. [22] and *Onchocerca* spp. (interspecific distances > 4.5%) suggested by Lefoulon et al. [5]. Interestingly, comparing sequences of *cox1* and 12S rRNA genes between five filarial nematodes recovered from species in the *S*. *asakoae* species-group and *S.* sp., and other filarioid nematodes suggested that they all represent the same new species. The phylogenetic trees of both genes showed that our filarial nematodes were separated from other filarioid nematodes, including the cryptic *Onchocerca* species infecting cervids in the USA [23] and the recently identified *Onchocerca* species, *O. borneensis*, which was discovered from the Bornean bearded pig (*Sus barbatus*) in Malaysia by Uni et al. [6].

We also found that the positive female of *S.* sp. in the *S*. *varicorne* species-group (BPD1) was most likely to be a new black fly species belonging to the *S*. *chumpornense* subgroup. Due to the limited sample size, further taxonomic and molecular analyses are needed to clarify the species status of this female.

Due to the morphological similarities of the *S. asakoae* species-group, which may lead to misidentification, we determined the species status of our positive samples belonging to this species-group based on an analysis of the *cox1* gene sequences. Two positive samples (BPD2 and BPD6) were true *S. asakoae*, supported by their genetic divergence that fell into the range of intraspecific divergence described for *S. asakoae* (0–2.27%) [24]. Besides *S. asakoae*, our positive samples seemed to be comprised of three known species (*S. monglaense*, *S. myanmarense*, and *S. tanahrataense*) and one unknown species in the *S. asakoae* species-group. Similar to the recent study of Jomkumsing et al. [25], who recorded *S. monglaense* and *S. myanmarense* from Thailand based on *cox1* gene sequences, we tentatively identified the distribution of these two black fly species in Chiang Mai province, northern Thailand, based on the same gene sequences. Additionally, based on *cox1* gene sequences, our study is the first to record *S. tanahrataense* in Thailand, although the species identification is only supported by molecular data (very low genetic divergence (0.18%) between our sample and true *S. tanahrataense*, and relatively high divergences (4.23–7.75%) with 10 other known species of the *S. asakoae* species-group). We also suggest that two positive samples (BPD8 and BPD9) are a distinct species near *S*. *brinchangense* in the *S. asakoae* species-group, although the genetic divergence between this new species and *S*. *brinchangense* was only 2.35–2.73%, which is less than the 3.0% proposed as cutoff genetic divergence for delimiting species boundaries in black flies [26,27,28]. When using a single gene, such as *cox1*, for black fly species identification, one has to be careful because of possible misidentification. In some cases, *cox1* gene sequences failed to differentiate two closely related species of the *S*. *asakoae* species-group. For example, the *cox1* gene sequences of *S*. *udomi* and *S*. *rampae* were almost identical and the genetic divergence between these two species was only 0.71–0.89% (Table S2). Therefore, in order to definitely conclude that these black fly species are actually present in Thailand, an integrated approach of morphotaxonomic and multi-locus sequence analyses is required.

Three different taxa of non-filarial nematodes classified by morphological characters were also found to infect three species of the *S. asakoae* species-group. The first taxon is molecularly similar to *I. lairdi*, which is a mermithid nematode. Although a small genetic distance (1.84%) was observed between our specimen and *I. lairdi*, we regard it as a distinct species based on the combination of morphological and molecular data. The low genetic differentiation observed was not unexpected, since the 18S rRNA gene is generally highly conserved [29]. For example, the variation of 18S rRNA gene sequences between different *Romanomermis* spp., the entomopathogenic nematodes of mosquito larvae, was only 0.4% [30]. In Thailand, only one publication has reported mermithid nematodes in black flies [31]. The investigators demonstrated that the larvae of five black fly species (*S. chiangmaiense*, *S. fenestratum*, *S. nakhonense*, *S. quinquestriatum*, and *S. tani*) were infected with single or multiple mermithid nematodes. However, the species of those mermithid nematodes had never been determined based on molecular data until the present study. The second taxon is most likely to be an unrecognized species belonging to the family Ascarididae and separated from all other ascaridoid nematodes in the 18S rRNA phylogenetic tree. Also considering the ornithophilic behavior of the *S. asakoae* species-group [9,12], it is probable that this worm belongs to the genus *Porrocaecum*, species of which are reported worldwide as common bird parasites [32]. As in mermithid nematodes, a low interspecific variation of the 18S locus was also observed between our specimen and several species of ascaridoid nematodes. More variable gene regions, which were highly effective for differentiating between closely related species, such as *cox1*, *cox2*, or *ITS1*, are required for resolving the taxonomic relationship between these nematodes [33]. The third taxon seems to be an unrecognized rhabditida nematode of the class Chromadorea based on 12S rRNA gene sequence data. Nevertheless, more molecular data are needed as the 12S rRNA phylogenetic tree revealed an unclear taxonomic relationship for this worm. More conserved gene regions, such as the 18S rRNA gene, which we unfortunately failed to amplify for this specimen, should be useful for resolving more distant taxonomic relationships [33]. Additionally, the infection rates of filarial and non-filarial nematodes in the *S. asakoae* species-group were similar to a previous study, in which at least two different species of non-filarial nematodes were found [10,12].

Apart from avian species (birds and chickens), suidae might also be a reservoir host of these filarial and non-filarial nematodes, because the black fly vectors in the *S. asakoae* species-group were collected in a pig farming area and swine (*Sus scrofa domesticus*) was also used as bait. Therefore, further studies to search for the adult stages of our parasites in the definitive host are needed.

Interestingly, females of *S. chumpornense* were collected in an extremely high number in the hot season (March) in both study areas, but rapidly disappeared after that. We assume that the outbreak of this black fly species may have been the result of the high temperature in the hot season, as well as a forest fire near the study areas during the black fly collections. These factors may affect the habitat and/or host-seeking behavior of this black fly species.

## 4. Materials and Methods

### 4.1. Ethics Statement

The protocol of this study was approved by the Research Ethics Committee (Institutional Animal Care and Use Committee) (Protocol Number 46/2561) and the Institutional Biosafety Committee (Approval No. CMUIBC02019/2562) of the Faculty of Medicine, Chiang Mai University, Chiang Mai province, Thailand.

### 4.2. Study Areas and Adult Female Black Fly Collections

**Figure 6:**
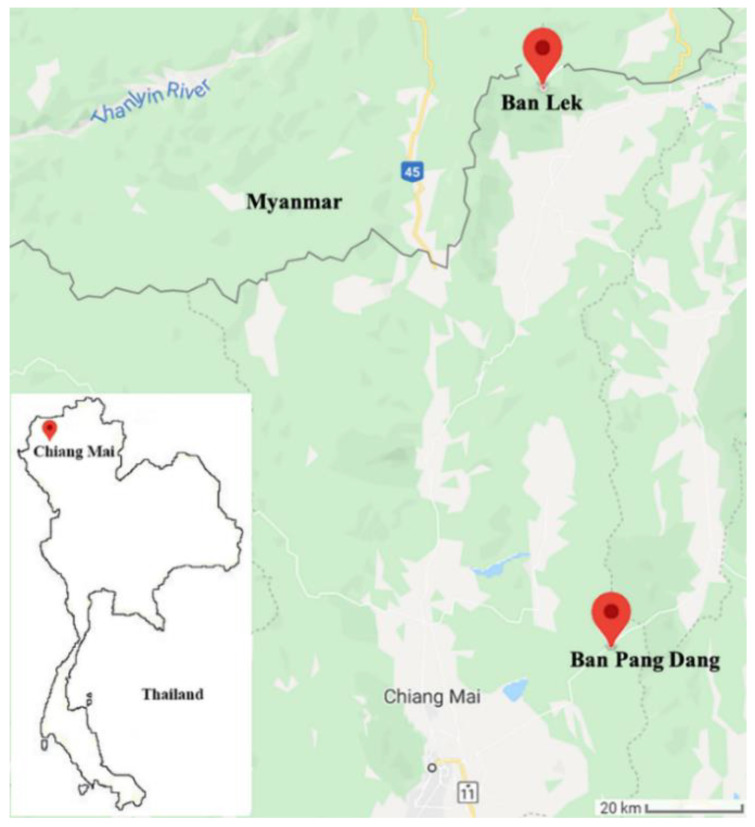
Map of two study areas in Chiang Mai province, northern Thailand, where black fly samples were collected.

Adult black fly collections were carried out at two selected sites in Chiang Mai province, northern Thailand (Figure 6). The first selected site was an open grassland located in Ban Lek village (20° 04′36.3′′ N 99°10′53.0′′ E, 1571 m in elevation), Fang district. The second selected site was in a pig farming area situated in Ban Pang Dang, Doi Saket district (19°01′30.9′′ N 99°19′02.7′′ E, 673 m in elevation).

Female adult black flies were captured two times each seasonally from March 2018 to January 2019 (hot-dry: March–June; rainy: August–October; cool-dry: November–January) by using a sweep net while flying around each human or animal (swine) bait. Each collection lasted for 2 h from 7.00–9.00 h in the early morning, which is a period of time in which most black fly species prefer to seek blood meal sources and attract hosts. They were kept in a paper cup with a pad of cotton wool soaked with 10% sucrose solution placed on the top-screen, and then stored in a humid chamber. After being transported to the Department of Parasitology, Faculty of Medicine, Chiang Mai University, Chiang Mai province, Thailand, they were morphologically identified, before being dissected and examined for filarial larvae.

### 4.3. Morphological and Molecular Identification of Female Black Flies

Adult female black flies caught were knocked out on ice for approximately 10 min and morphologically identified under a stereomicroscope using the standard keys of Takaoka et al. [34]. In addition, DNA barcoding based on the *cox1* gene was conducted to confirm the species of the infected flies. In brief, the total DNA of individual files was extracted using the PureLink^®^ Genomic DNA Mini Kit (Invitrogen, Carlsbad, CA, USA). The mitochondrial *cox1* gene was amplified using a universal primer set: LCO1490 (5′-GGT CAA CAA ATC ATA AAG ATA TTG G-3′) and HCO2198 (5′-TAA ACT TCA GGG TGA CCA AAA AAT CA) [35]. DNA amplification was carried out with the reaction mixture (20 µL) containing 1–2 µL of DNA template, 0.5 U of *Taq* DNA polymerase, 3 mM of MgCl_2_, 0.25 mM dNTPs, and 0.2 µM of each primer under the PCR cycling parameters previously described by Conflitti et al. [36]. PCR fragments were visualized on 1.5% agarose gels stained with SYBR Safe DNA gel stain (Invitrogen, Carlsbad, CA, USA). The PCR products were then sent to Macrogen, Inc. (Seoul, Korea) to purify and sequence them in both directions using the same primers as in PCR by a 3130 genetic analyzer (Applied Biosystems, Foster, CA, USA).

Both forward and reverse sequences were assembled and edited manually in the MEGA Version 7.0 program [37]. Sequence alignment was performed using the ClustalW multiple alignment programs [38]. Gap sites were treated as missing data for the following analyses.

Phylogenetic trees based on the *cox1* gene sequences were constructed using neighbor-joining (NJ), maximum-likelihood (ML), and Bayesian inference (BI) methods. The best-fit models for ML and BI methods were selected using the jModelTest version 2.1.10 program [39]. The NJ tree was built with the MEGA version 7.0 program [37]. Bootstrap values for supporting each clade node were calculated using the bootstrapping method with 1000 replicates. The ML tree was developed using PhyML 3.3 with 100 bootstrap replicates [40]. BI was performed in MrBayes v.3.2.7 [41] and run for at least two million generations or until the ASDSF was less than 0.01 with sampling every 100 generations and a burnin of 25%. Genetic divergences within and between species were estimated using Kimura’s two-parameter (K2P) model [42]. The DNA sequence of *Prosimulium mixtum* was used as the outgroup for all analyses.

### 4.4. Dissections of Black Flies and Morphological and Molecular Identification of Recovered Larvae

After species identification, individual flies were dissected to search for filarial larvae on a glass slide in a drop of PBS pH 7.4 or 0.9% NSS under a stereomicroscope at 10× magnification. The unknown larvae recovered were observed, measured, and identified to a genus level following the identification key of Bain and Chabaud [17] under a compound microscope, Olympus BX53 (Olympus, Shinjuku, TYO, Japan). Subsequently, they were preserved in 80% ethanol and kept at −20 °C until being used for molecular analysis. Descriptive statistics were used to calculate the percentage of infection rate.

The unknown larvae recovered were molecularly identified using mitochondrial *cox1* and 12S rRNA genes. In some cases, in which the amplification of both mitochondrial genes failed, the nuclear 18S rRNA (*SSU* HVR-I) gene was used instead. The specimens of *Onchocerca* sp. type I (*n* = 6) recovered from *S. nigrogilvum* from our preliminary study were also included for molecular analysis. Genomic DNA of individual larvae was extracted using the PureLink^®^ Genomic DNA Mini Kit (Invitrogen, Carlsbad, CA, USA). PCR amplification was conducted by using the primers and annealing temperatures listed in Table 4 with the reaction mixture of 20 µL, consisting of 1–2 µL of DNA template, 0.5 U of *Taq* DNA polymerase, 2–3 mM of MgCl_2_, 0.25 mM dNTPs, and 0.2 µM of each primer under PCR cycling conditions described elsewhere [15,43]. The amplified products were purified and sequenced following the methods mentioned in Section 4.3.

After DNA sequencing, the aligned *cox1*, 12S rRNA, and 18S rRNA gene sequences of the filarial and non-filarial larvae were used to construct the phylogenetic trees separately for each gene using the procedures described in Section 4.3. *Thelazia callipaeda* was used as the outgroup species for all analyses of the filarial nematodes. For an 18S rRNA phylogenetic tree of mermithid and ascaridoid nematodes, *Clarkus papillatus* and *Brugia malay* and *Physaloptera mirandai* were used as the outgroup species for all analyses, respectively. Moreover, *Heterorhabditis bacteriophora* and *Heterorhabditis indica* were used as the outgroup species for all analyses of the 12S rRNA phylogenetic tree of rhabditida nematodes. For comparison, selected published sequences with the corresponding accession numbers listed in Figure 2, Figure 3 and Figure 4 were included in the analysis.

Additionally, all of the sequences generated were compared with available sequences deposited in GenBank using the Basic Local Alignment Search Tool (BLAST) and were registered in the National Center for Biotechnology Information (NCBI) GenBank database under the following accession numbers: MT262563-MT262591, MT262493-MT262509, MT261052-MT261068, and MT396080-MT396082.

## 5. Conclusions

We discovered an unknown filarial species (probably a new species) in *S. asakoae*, *S. myanmarense,* and *S. monglaense* (all genetically identified) in the *S. asakoae* species-group, and *S*. sp. in the *S. varicorne* species-group, and also confirmed the presence of *O*. sp. type I in *S*. *nigrogilvum* collected in the Chiang Mai province, Thailand. In addition, three species of unknown non-filarial nematodes were found in *S*. *asakoae*, *S. monglaense*, and *S*. sp. in the *S. asakoae* species-group. Due to the limited sample size and ambiguous species status of these non-filarial larvae, further study is required to clarify whether they have the potential to be pathogens to humans. Unidentified nematodes were also obtained from *S*. *tanahrataense* and *S*. sp. in the *S*. *asakoae* species-group.

The fact that *S*. *nigrogilvum* and species in the *S*. *asakoae* species-group are anthropophilic and natural vectors of filarial worms, though their infection rates were low, suggests that people who visit or live in these areas are at risk of zoonotic filarial infections, particularly during the hot and rainy seasons.

## Figures and Tables

**Table 1:** Seasonal abundance and natural filarial infections of female black flies collected at Ban Lek, Fang district, Chiang Mai province, northern Thailand, from March 2018 to January 2019.

Season	Month	*Simulium* Species	No. Collected		No. Infected	No. L_3_	% Infected
Hot	March	*S. chumpornense*	317	477	0	0	0
		*S*. *asakoae* species-group	111		0	0	0
		*S. nigrogilvum*	36		1	1	2.78
		*S. striatum* species-group	13		0	0	0
	May	*S. nigrogilvum*	151	153	2	3	1.32
		*S. chamlongi*	1		0	0	0
		*S. doipuiense* complex	1		0	0	0
Rainy	August	*S. nigrogilvum*	34	48	0	0	0
		*S*. *asakoae* species-group	13		0	0	0
		*S. striatum* species-group	1		0	0	0
	October	*S. nigrogilvum*	48	65	1	2	2.08
		*S*. *asakoae* species-group	14		0	0	0
		*S. manooni*	1		0	0	0
		*S. doipuiense* complex	1		0	0	0
		*S. striatum* species-group	1		0	0	0
Dry-cool	December	*S. nigrogilvum*	40	59	0	0	0
		*S. doipuiense* complex	10		0	0	0
		*S*. *asakoae* species-group	7		0	0	0
		*S. chamlongi*	1		0	0	0
		*S. striatum* species-group	1		0	0	0
	January	*S*. *asakoae* species-group	3	3	0	0	0
		Total	805		4	6	-

Abbreviation: L_3_ = third-stage larva (infective larva).

**Table 2:** Seasonal abundance and natural nematode infections of female black flies collected at Ban Pang Dang, Doi Saket District, Chiang Mai province, northern Thailand, from March 2018 to January 2019.

Season	Month	*Simulium* Species	No. Collected		No. Infected	No. Larva			% Infected
						FL	NFL	UID	
Hot	March	*S. chumpornense*	2249	2616	0	0	0	0	0
		*S*. *asakoae* species-group	363		0	0	0	0	0
		*S. nigrogilvum*	3		0	0	0	0	0
		*S.* sp.	1		1	1 L_1_	0	0	100
	June	*S*. *asakoae* species-group	157	157	1	0	0	1 Mf	0.64
Rainy *	September	*S*. *asakoae* species-group	635	636	2	1 L_3_	1 L_3_	0	0.31
		*S. striatum* species-group	1		0	0	0	0	0
Dry-cool	November	*S*. *asakoae* species-group	368	368	3	2 L_3_	1 L_3_	1 L_1_	0.82
	January	*S*. *asakoae* species-group	807	820	2	1 L_3_	1 L_3_	0	0.25
		*S. striatum* species-group	8		0	0	0	0	0
		*S. chamlongi*	2		0	0	0	0	0
		*S. tani*	1		0	0	0	0	0
		*S. bullatum*	1		0	0	0	0	0
		*S. lampangense*	1		0	0	0	0	0
		Total	4597		9	5	3	2	-

Abbreviations: Mf = microfilaria; L_1_ = first-stage larva; L_3_ = third-stage larva (infective larva); FL = filarial nematode; NFL = non-filarial nematode; UID = unidentified nematode; * black fly collections were conducted two times in this season.

**Table 3:** Measurements, stages, and molecular identification of filarial and non-filarial larvae recovered from adult female black flies collected from Ban Lek and Ban Pang Dang, Chiang Mai province, northern Thailand.

Black Fly Species (No.).	Larval Stage (No.)	Body Length × Body Width (μm)	Molecular Identification
Filarial nematodes *S. nigrogilvum* (4) *S. asakoae* species-group (3) *Simulium* sp. (1)	L_3_ (6) L_3_ (4) L_1_ (1)	(1027.1–1339.5) × (25.6–29.6) (576.1–688.8) × (26.9–28.6) 270.3 × 18.6	^1^*Onchocerca* sp. type I ^1^ Filarioid nematode ^1^ Filarioid nematode
Non-filarial nematodes *S. asakoae* species-group (3)	L_3_ (1) L_3_ (1) L_3_ (1)	2864.1 × 102.7 446.4 × 20.3 606.2 × 26.9	^3^ Mermithid nematode ^2^ Rhabditida nematode ^3^ Ascaridoid nematode
Unidentified nematodes *S*. *asakoae* species-group (2)	Mf (1) L_1_ (1)	336.6 × 9.6 468.9 × 16.2	N/A

^1^ Based on *cox1* and 12S genes; ^2^ based on the 12S rRNA gene; ^3^ based on the 18S rRNA gene. Abbreviations: Mf = microfilaria; L_1_ = first-stage larva; L_3_ = third-stage larva (infective larva); N/A = not available.

**Table 4:** Details of primers used for amplifying mitochondrial and nuclear genes of recovered larvae.

Region Amplified	Primer		Sequence (5′-3′)	Annealing T (°C)	Product (bp)	Ref
*cox1*	Fw	COIintF	TGATTGGTGGTTTTGGTAA	48	~689	[44]
	Rev	COIintR	ATAAGTACGAGTATCAATATC			
12S rRNA	Fw	12SF	GTTCCAGAATAATCGGCT	50	~502	[45]
	Rev	12SR	ATTGACGGATGRTTTGTACC			
18S rRNA (*SSU* HVR-I)	Fw	RS5401	AAAGATTAAGCCATGCATG	50	~919	[43]
	Rev	RS5402	CATTCTTGGCAAATGCTTTCG			

Abbreviations: Fw = forward primer; Rev = reverse primer; T = temperature; Ref = reference.

## Supplementary Materials

The following are available online at https://www.mdpi.com/2076-0817/9/6/512/s1: Table S1: NCBI BLAST results of filarial and non-filarial nematodes recovered from *Simulium* spp. based on mitochondrial *cox1*, 12S rRNA, and nuclear 18S rRNA (*SSU* HVR-I) gene sequences; Table S2: Range of interspecific genetic divergence (%) among the infected black flies belonging to the *S. asakoae* species-group.
