# Supplementary material for: Seasonal Filarial Infections and Their Black Fly Vectors in Chiang Mai Province, Northern Thailand
Source: Pathogens. 2020 Jun 25;9(6):512. doi: 10.3390/pathogens9060512 (PMC7350311; doi:10.3390/pathogens9060512)
Supplement: Supplementary file 1 [file pathogens-09-00512-s001.pdf]

Table S1. NCBI BLAST results of filarial and non-filarial nematodes recovered from *Simulium* spp. based on mitochondrial *cox1*, 12S rRNA and nuclear 18S rRNA (*SSU* HVR-I) gene sequences.

Black fly species	Specimen Isolate	Gene	Closest species	% similarity	GenBank accession number
S. sp.	BPD1	*Cox1*	*Dirofilaria repens*	90	MH780816
			Loa loa		HQ186250
			Onchocerca gibsoni		AJ271616
		12S rRNA	*Wuchereria bancrafti*	89	JF775522
			Brugia malayi		AF538716
			Loa loa		HQ186250
S. asakoae species-group	BPD2, BPD3, BPD4.1, BPD4.2	*Cox1*	*Dirofilaria repens*	90	MH780816
			Loa loa		HQ186250
			Onchocerca gibsoni		AJ271616
		12S rRNA	*Wuchereria bancrafti*	88-90	JF775522
			Brugia malayi		AF538716
			Loa loa		HQ186250, AJ544845, FR827907
	BPD5	18S rRNA	*Isomermis lairdi*	98	FN400900
	BPD6	12S rRNA	*Litoditis aff. Marina PmIV*	91	KR815453
	BPD8	18S rRNA	*Porrocaecum streperae*	98	EF180074
			Pseudoterranova decipiens		U94380
			Baylisascaris procyonis		U94368
			Ascaris suum		U94367
S. nigrogilvum	BL1, BL2, BL3.1, BL3.2, BL4.1, BL4.2, BL5.1, BL5.2, BL5.3, BL5.4, BL5.5, BL6	*Cox1*	*Onchocerca* sp. type A	99-100	AB518876
		12S rRNA		100	AB518877, AB518878, AB518879

Table S2. Range of interspecific genetic divergence (%) among the infected black flies belonging to *S. asakoae* species-group.

	**Black fly species**	**1**	**2**	**3**	**4**	**5**	**6**	**7**	**8**	**9**	**10**	**11**	**12**	**13**	**14**	**15**	**16**	**17**	**18**	**19**
**1**	*S.* sp. BPD2																			
**2**	*S.* sp. BPD3	8.93																		
**3**	*S.* sp. BPD4	8.53	2.73																	
**4**	*S.* sp. BPD5	9.15	2.92	1.62																
**5**	*S.* sp. BPD6	0.54	8.73	8.33	8.95															
**6**	*S.* sp. BPD7	7.75	4.42	4.43	3.28	7.76														
**7**	*S.* sp. BPD8	7.53	6.74	6.15	5.95	7.75	5.55													
**8**	*S.* sp. BPD9	7.53	6.74	6.15	5.95	7.75	5.55	0												
**9**	*S. asakoae*	0.00-2.17	8.32-8.93	7.92-8.53	8.13-9.15	0.54-1.99	6.95-7.75	7.34-7.53	7.34-7.53											
**10**	*S. monglaense*	8.52-8.73	2.92-3.48	0.18-2.17	1.62-1.81	8.54-8.73	4.23-4.62	6.14-6.35	6.14-6.35	7.91-8.73										
**11**	*S. myanmarense*	8.93-9.14	0.00-0.18	2.73-2.92	2.92-3.11	8.73-8.94	4.42-4.61	6.74-6.94	6.74-6.94	8.32-9.14	2.92-3.67									
**12**	*S. lurauense*	9.15-9.56	8.49-8.90	8.70-9.11	7.69-8.09	9.34-9.76	6.71-7.11	7.92-8.33	7.92-8.33	8.52-9.56	7.88-9.32	8.49-9.10								
**13**	*S. sofiani*	9.35	8.49	8.7	8.09	9.55	7.11	8.33	8.33	8.72-9.35	7.88-8.90	8.49-8.69	0.36-0.54							
**14**	*S. udomi*	8.37	6.75	6.36	6.56	8.18	7.76	6.79	6.79	7.36-8.37	6.56-7.36	6.75-6.96	8.52	8.93						
**15**	*S. rampae*	8.38-8.59	6.56-6.76	5.78-5.97	5.97-6.17	8.19-8.40	7.36-7.56	6.60-6.40	6.60-6.40	7.37-8.59	6.97-6.96	6.56-6.96	7.91-8.12	8.32-8.53	0.71-0.89					
**16**	*S. brinchangense*	7.36	7.53-7.74	6.74-6.94	6.54-6.74	7.57	5.57-5.76	2.35-2.73	2.35-2.73	7.17-7.57	6.73-7.14	7.53-7.94	7.54-7.95	7.95	7.39	7.20-7.40				
**17**	*S. tanahrataense*	7.95	4.61	4.62	3.47	7.96	0.18	5.74	5.74	7.15-7.95	4.42-4.81	4.61-4.81	6.91-7.31	7.31	7.96	7.56-7.77	5.76-5.96			
**18**	*S. izuae*	9.35	3.30-3.49	2.36-2.93	2.18-2.36	9.16	4.23-4.43	5.95-6.15	5.95-6.15	7.52-9.35	2.55-3.68	3.30-3.68	8.49-9.11	8.90-9.11	6.76-6.96	6.57-6.97	6.34-6.74	4.43-4.62		
**19**	*S. roslihashimi*	8.95-9.16	3.29-3.67	2.36	1.81	8.75-8.96	4.61-4.81	5.76-6.16	5.76-6.16	7.12-9.16	2.55-3.10	3.29-3.86	8.30-8.91	8.71-8.91	6.57-6.97	6.38-6.58	6.15-6.55	4.81-5.00	1.62-2.17	
